# The Drosophila *gypsy* Insulator Supports Transvection in the Presence of the *vestigial* Enhancer

**DOI:** 10.1371/journal.pone.0081331

**Published:** 2013-11-13

**Authors:** Todd Schoborg, Srilalitha Kuruganti, Ryan Rickels, Mariano Labrador

**Affiliations:** Department of Biochemistry and Cellular and Molecular Biology, University of Tennessee, Knoxville, Tennessee, United States of America; University of Crete, Greece

## Abstract

Though operationally defined as *cis-*regulatory elements, enhancers can also communicate with promoters on a separate homolog in *trans*, a mechanism that has been suggested to account for the ability of certain alleles of the same gene to complement one another in a process otherwise known as transvection. This homolog-pairing dependent process is facilitated in Drosophila by chromatin-associated pairing proteins, many of which remain unknown and their mechanism of action uncharacterized. Here we have tested the role of the *gypsy* chromatin insulator in facilitating pairing and communication between enhancers and promoters in *trans* using a transgenic *eGFP* reporter system engineered to allow for targeted deletions in the *vestigial* Boundary Enhancer (*vg*BE) and the *hsp70* minimal promoter, along with one or two flanking *gypsy* elements. We found a modest 2.5-3x increase in *eGFP* reporter levels from homozygotes carrying an intact copy of the reporter on each homolog compared to unpaired hemizygotes, although this behavior was independent of *gypsy*. However, detectable levels of GFP protein along the DV wing boundary in trans-heterozygotes lacking a single enhancer and promoter was only observed in the presence of two flanking *gypsy* elements. Our results demonstrate that *gypsy* can stimulate enhancer-promoter communication in *trans* throughout the genome in a context-dependent manner, likely through modulation of local chromatin dynamics once pairing has been established by other elements and highlights chromatin structure as the master regulator of this phenomenon.

## Introduction

Unlocking the mechanism by which gene regulatory elements (enhancers, promoters and other transcription factor binding sites) coordinate gene expression in a precise spatio-temporal manner is critical to understanding how eukaryotic genomes function *in vivo*. Chromatin structure plays a central role in this process, where nucleosome position and density, chromatin insulators, histone modifications and their associated proteins function to modulate the properties of these regulatory elements. Most of the effort in understanding this interplay has focused on their behavior in *cis—*that is, interactions occurring along the same chromosome. Indeed, enhancers are defined as *cis-*regulatory elements that function to stimulate gene expression when located either distal or proximal to their cognate promoters, and can function over large distances [1-4]. This is achieved through physical association between the enhancer and the promoter, mediated by a number of regulatory proteins, general transcription factors, RNA Pol II and chromatin binding proteins that result in the formation of chromatin loop structures that are critical for transcriptional activation [5-9].

However, the ability of enhancers to also act in *trans* (i.e., on a separate DNA molecule) on promoters has been observed both *in vitro* and *in vivo* [7,10-14] where such behavior has been suggested to account for a number of homolog pairing-dependent phenotypes, such as in the phenomenon of transvection (see 15-19 for review). The term transvection was first coined by E.B. Lewis in 1954 to describe the ability of certain Drosophila alleles of *Ultrabithorax* (*Ubx*) to complement one another, leading to partial rescue of the mutant phenotype [20]. Importantly, this rescue failed when the locus on either homolog was relocated to a new position on the chromosome, suggesting that somatic pairing between homologous chromosomes is essential for transvection. This type of intragenic complementation has been reported almost exclusively in Drosophila, with transvection effects observed at a number of loci: *yellow* (y) [21,22], *decapentaplegic* (*dpp*) [23], *eyes absent* (eya) [24], *white* (w) [25], *Gpdh* [26], *hedgehog* (*hh*) [27], *wingless* (*wg*) [28], *engrailed (en*) [29], *pointed* (*pnt*) [30], *cubitus interrruptus* (*ci*) [31], *sex combs reduced (scr*) [32], *brown* (*bw*) [33,34] and *vestigial* (*vg*) [35]. Recent studies have concluded that transvection is pervasive throughout the entire Drosophila genome [11,36], a finding not surprising given that homologs remain paired in somatic nuclei during interphase in flies [37]. In other eukaryotes, homologs in somatic tissue do not remain in close synapse throughout interphase, yet quite a few cases of transvection and other pairing dependent phenomenon have been reported in model systems other than Drosophila, including yeast [38], plants [39] and mammals [40-42], suggesting that eukaryotes possess evolutionarily conserved mechanisms that allow homologous chromosomes to communicate in *trans.*

Given the need for physical associations between enhancers and promoters to generate a sustained transcriptional output, homolog pairing in *trans* can facilitate contacts between a functional enhancer located on one homolog and a functional promoter located on the other, increasing the frequency of collisions between the two elements and thus the probability that a stable ternary complex is established. A number of proteins have been shown to be required for homolog pairing in Drosophila meiosis, including the multi-subunit cohesin complex (SMC1, SMC3, SCC1/RAD21, SCC3), although it does not appear to be required for somatic paring [43]. In mammals, this complex has been shown to be involved in stable *cis* looping contacts between enhancers and promoters, suggesting it may play a more direct role in gene regulation [44-46]. Only a handful of Drosophila genes, mainly involved in mitotic functions, cell cycle control and chromatin organization, including *Topoisomerase II* (*Top2*) have been shown to promote somatic pairing [43,47], although other chromatin binding proteins, such as Zeste and members of the Polycomb Group Complex, have been shown to be required for transvection in a number of cases, including those involving communication between enhancers and promoters in *trans* [25,32,48-52]. 

In theory, however, any DNA element and its associated proteins that can mediate stable long-range contacts between distant genomic sites could potentially function to stabilize homolog pairing to facilitate enhancer-promoter interactions in *trans*. Chromatin insulators are well-suited for this task, given their ability to mediate long-distance contacts along the chromatin fiber in vivo. These DNA elements were first identified in Drosophila based on their ability to block enhancer-promoter communication and heterochromatin spreading along the chromatin fiber in transgenic assays. Such properties are conferred by insulator-binding proteins, seven of which have been characterized in Drosophila, including Su(Hw), CP190, BEAF-32, Mod(mdg4)67.2, dCTCF, GAF and Zw5, with mammals containing only a CTCF ortholog highly divergent in amino acid similarity to its Drosophila counterpart [53]. The *gypsy* insulator located within the 5' LTR of the *gypsy* retrotransposon is perhaps the most well characterized insulator, whose enhancer and heterochromatin blocking properties are conferred by Su(Hw), CP190 and Mod(mdg4)67.2. However, in addition to *gypsy* there are thousands of endogenous insulator sites located throughout the genome, where combinatorial binding of insulator proteins to each of these sites suggests a complex landscape whose functional consequences remain poorly understood [54-56]. Recent work has shown that these elements help mediate long-range contacts between enhancers, promoters and other insulator sites in order to direct transcriptional outputs, maintain regions of histone modifications, and establish gene regulatory and physical domains [2,57-60].

Though most of these interactions are thought to occur in *cis*, potential interactions in *trans* are not out of the question, even for long range contacts between non-homologous sites, analogous to the behavior observed for olfactory receptor choice by the *H* enhancer and various *olfactory* gene promoters located on different chromosomes in mice [61]. Interestingly, *gypsy* insulators have been previously implicated in transvection, both directly and indirectly, at the *yellow* locus [21,62]. Additionally, the *bx* ^*34e*^ allele, used by E.B. Lewis in his original description of the phenomenon [20], results from a *gypsy* retrotransposon insertion between the *bx* enhancer and the promoter of *Ubx* and subsequent loss of enhancer-promoter communication in *cis* due to the enhancer blocking property of the *gypsy* insulator. Furthermore, reduction of Su(Hw) has been shown to reduce somatic pairing by ~30% in embryos [63], suggesting that insulators might contribute to pairing dependent enhancer-promoter communication in *trans.*

Here we have used a reporter construct designed to elucidate the role of the *gypsy* insulator in transvection. We engineered our system with the *vestigial* (*vg*) boundary enhancer (*vg*BE) and a minimal hsp70 promoter to drive *eGFP* expression, flanked by a *gypsy* insulator upstream and downstream (2-insulator), upstream only (1-insulator) or no *gypsy* insulator (0-insulator). Using the Cre/*loxP* and Flp/*FRT* system to delete the promoter or enhancer, respectively, “promoterless” and “enhancerless” flies were created and crossed to measure transvection effects. Quantitative fluorescent microscopy and qPCR of wing discs from 3^rd^ instar larvae reveal a pairing dependence in the non-deletion constructs that is independent of *gypsy*, whereas transvection was only visually observed along the DV wing boundary in lines containing two flanking insulators. Interestingly, the *vg*BE alone can drive expression of a large pool of e*GFP* transcripts in *cis* in the absence of a functional promoter, the majority of which are not translated into protein. Taken collectively, our results demonstrate that the *gypsy* insulator can contribute to transvection in a dose-dependent manner, likely through modulation of local chromatin dynamics once other chromatin elements have established homolog pairing. 

## Materials and Methods

### Fly Stocks & Husbandry

Flies were cultured on standard cornmeal-agar media and maintained at 25°C. Flippase (FLP) (*y* ^*1*^ *w* ^1118^ *P*{*ry[+t7.2*]*=hsFLP*}*1; DrMio*/TM3*, ry* ^*^ *Sb* ^*1*^), Cre recombinase (*y* ^*1*^ *w* ^67c23^; *nocSco*/CyO*, P*{*w* ^*+mC*^ *=Crew*}*DH1*) and *su*(*Hw*) mutant flies (*w* ^*1118*^ *; PBac{RB*}*Su(Hw*)*^e04061^/TM6B, Tb* ^*1*^) were obtained from the Bloomington Stock Center. Microinjection to generate 2-insulator (*yw; P{Ins-vgBE-eGFP-Ins, w* ^*+mC*^}), 1-insulator (*yw; P{Ins-vgBE-eGFP, w* ^*+mC*^}) and 0-insulator (*yw; P{vgBE-eGFP, w* ^*+mC*^}) transgenics was performed by Genetivision (Houston, TX). The *w; nocSco*/CyO; MKRS, *Sb1*/TM6B*, Tb* ^*1*^ double balancer line was a gift from Bruce McKee.

### Construct Design and Plasmid Generation

**Figure 1:**
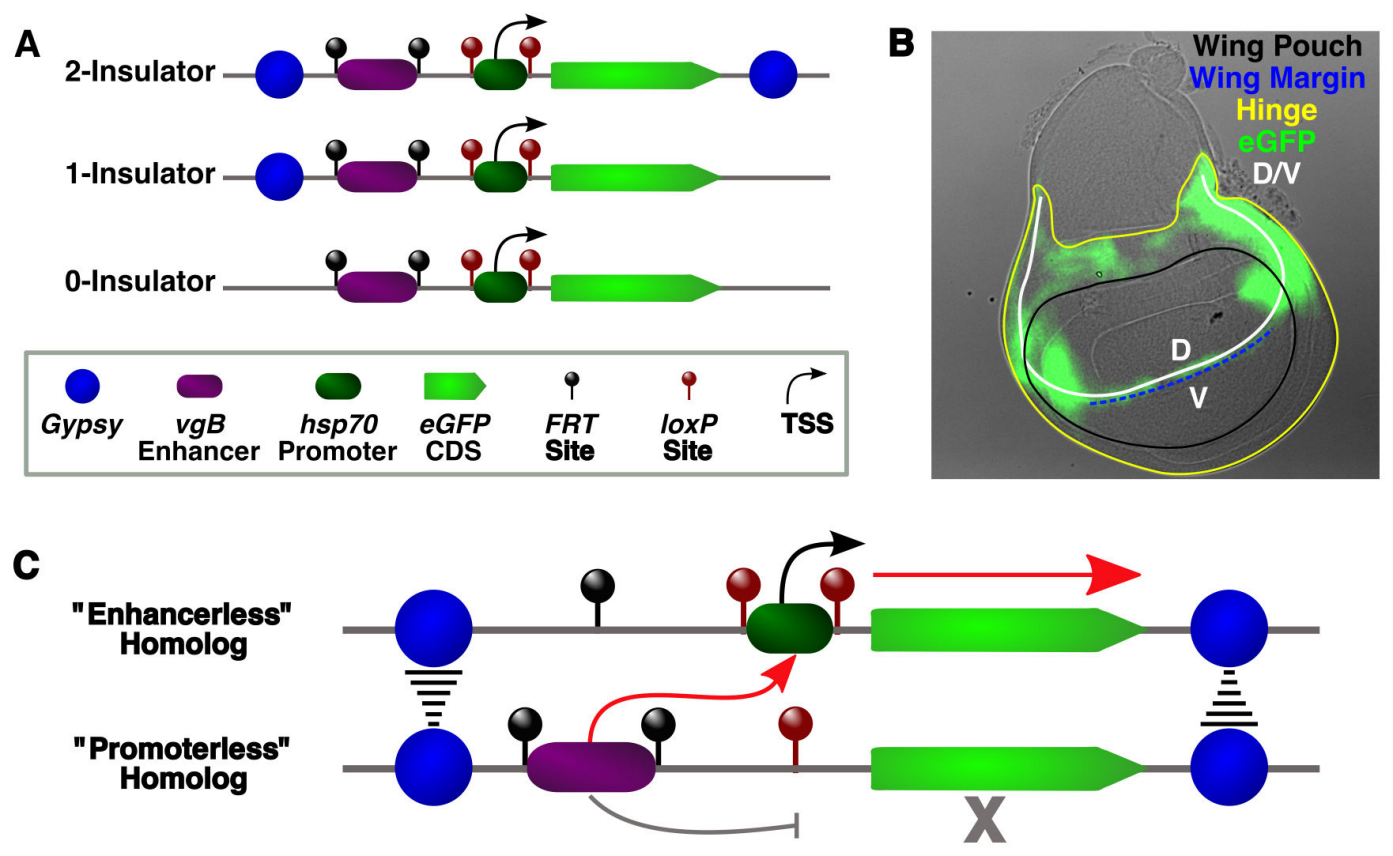
Schematic of the reporter system used in this study. All constructs contained the *vg*BE enhancer flanked by *FRT* sites, a minimal *hsp70* promoter flanked by *loxP* sites, an *eGFP* coding sequence, and either zero, one or two *gypsy* insulators (A). Differential Interference Contrast (DIC)-GFP overlay of a 3^rd^ instar wing disc showing the expression pattern of the *vg*BE along the dorsal-ventral (D/V) boundary, particularly within the hinge and wing margin (B). Communication in *trans* between a functional enhancer (“promoterless”) on one homolog and a functional promoter (“enhancerless”) on the other homolog might be facilitated by *trans* interactions between flanking *gypsy* insulators, leading to expression only from the “enhancerless” homolog (C).

The reporter construct consisted of 5 core elements: the *vestigial* boundary enhancer (*vg*BE), *loxP* and *FRT* sites, *5xUAS/hsp-70* minimal promoter, and an *eGFP* coding sequence. Additional *gypsy* insulator sequences were present as required to generate either the 1- or 2-insulator construct (Figure 1A). The pGREEN pelican plasmid, consisting of an eGFP reporter with a 5' MCS flanked by two *gypsy* insulators, served as the vector backbone for these constructs [64]. First, the *5xUAS/hsp-70* minimal promoter was amplified from the pUAST vector using specific primers designed to insert NheI and XhoI cut sites and a single *loxP* site oriented in the same direction on both sides of the promoter. The PCR product was digested with *Nhe*I and *Xho*I and cloned into the pGREEN pelican vector digested with *Nhe*I and *Xho*I to obtain the pGP-hsp70 plasmid. The wing and haltere disc *vestigial* Boundary Enhancer (*vg*BE) present in the second intron of the *vg* gene [65] was amplified from *yw* gDNA with primers engineered with BbvCI and BamHI sites, digested and cloned into pCR 2.1-λ- FRT that was available in the lab. This plasmid consists of λ DNA (~800bp) and *FRT* sequences in the same orientation flanking the multiple cloning site. The cloned *vg*BE enhancer and its flanking *FRT* sites were digested as a *Kpn*I-*Sac*II fragment and cloned into pGP-hsp70 to obtain the 2-insulator construct *Ins-VgBE-eGFP-Ins*. To obtain the 1-insulator construct (*Ins-VgBE-eGFP*), the 3' insulator downstream of eGFP was deleted from this vector by restriction digestion with *Spe*I and *Eco*47III. The sticky ends generated by *Spe*I digestion were end-filled using *Pfu* DNA polymerase and blunt ends were ligated. To generate the 0-insulator construct (*VgBE-eGFP*), both insulators were deleted from the pGP-hsp70 plasmid, generating a *Kpn*I-λ-*vg*BE-eGFP-*Sac*II cassette that was reinserted into the insulator-less pGP-hsp70 plasmid. All constructs were microinjected into *y* ^*1*^ *w* ^67c23^ flies and individual lines established by *w* ^+^ selection. 

### Insertion Mapping & Inverse PCR

Individual lines were mapped using both classical and molecular genetics. A single male homozygous for the transgene (*w* ^*+*^) from each line was crossed to *w; nocSco*/CyO; MKRS, *Sb1*/TM6B*, Tb* ^*1*^ virgins. Progeny males carrying *w* ^+^ *, CyO* and *TM6B* were then crossed to *yw* virgins and the resulting offspring scored to determine how *w* ^+^ segregated with respect to the dominantly-marked *CyO* and *TM6B* balancers. Inverse PCR to identify the precise insertion position in the genome was carried out as described (Berkley Drosophila Genome Project; http://www.fruitfly.org/about/methods/inverse.pcr.html). Genomic DNA was extracted from adult flies with DNAzol (Invitrogen), ~1μg digested with *Hin*PI*, Sau*3AI or *Msp*I in a 30 μl reaction volume for 3 hrs at 37°C and ligated (T4 DNA ligase, NEB) overnight at 4°C in a 400 μl reaction volume. DNA was EtOH precipitated and washed followed by resuspension in 10 μl H_2_O and amplified with both the *Pry1/Pry4* and *PwhtI/Plac1* primer sets. Samples producing a single strong band with minimum background were PCR purified and sequenced with either the *Sp1* or *Spep1* primer. Sequences were mapped to the latest version of the Drosophila genome using the BLAST algorithm at FlyBase. 

### Inducing Artificial Mutations in the Enhancer and Promoter

To delete the promoter, transgenic males carrying an intact construct were crossed with virgin females carrying Cre recombinase. Individual progeny males (*w* ^*+*^) were then crossed to *yw* virgins and gDNA extracted from adult progeny using DNAzol (Invitrogen), followed by PCR to screen for promoter removal. A similar crossing scheme was used to remove the *vg*BE enhancer, with transgenic males carrying an intact construct crossed with virgins carrying *flippase* under control of the *hsp-70* promoter. Larvae were subjected to daily heat shock by submerging vials in a 37°C water bath for 1 hr. Individual males (*w* ^*+*^) were then crossed to *yw* virgins and gDNA extracted from adult progeny to screen for enhancer removal by PCR. 

### Genotype Nomenclature

For each line, a total of seven genotypes were analyzed: (1) the intact construct containing enhancer and promoter homozygote (P^+^E^+^/P^+^E^+^); (2) the intact construct containing enhancer and promoter hemizygote (P^+^E^+^/+); (3) deleted “promoterless” homozygote (P^-^/P^-^); (4) deleted “promoterless” hemizygote (P^-^/+); (5) deleted *vg*BE “enhancerless” homozygote (E^-^/E^-^); (6) deleted *vg*BE “enhancerless” hemizygote (E^-^/+) and (7) Trans-heterozygote (transvection) (P^-^/E^-^), derived from crossing “promoterless” and “enhancerless” homozygotes. All hemizygotes were obtained by crossing the homozygote to *yw* or *y* ^*2*^ *wct* ^6^ *; su*(*Hw*)*e04061*/TM6B, *Tb^1^.*

### Immunostaining and Microscopy

Immunostaining was performed as described [66]. Wing imaginal discs were dissected from the late third instar larvae in SFX-Insect media (Hyclone) and fixed with 500µl Fixation Buffer (4% PFA/0.5%Triton/1xPBS) for 30 min at RT with rotation. The discs were washed 3X with Block-Permeabilization solution (1% BSA/0.5% Triton/1X PBS) for 10 min each, and then incubated in the same solution for 1 hr at RT with rotation. Discs were then incubated with α-GFP (Invitrogen) diluted 1:350 in wash solution (1% BSA/0.1% Triton/1X PBS) for 1 hr at RT. After washing 3X for 10 min each, discs were incubated with α-rabbit IgG-Texas Red secondary antibody (Jackson ImmunoResearch) at 1:500 for 1 hr at RT, washed 3X with PBST for 10 min each, counterstained with DAPI and rinsed with H_2_O. Discs were then oriented on coverslips containing Poly-L lysine and mounted in Vectashield. 

Images were obtained on a Lecia DM6000B widefield epifluorescent microscope equipped with a Hamamatsu ORCA-ER CCD camera and a HC PL FLUOTAR 20x/.50NA objective. Simple PCI (v6.6) was used for acquisition of raw images, which were processed using AutoQuant's 3D Deconvolution Algorithm utilizing an adaptive (blind) PSF implemented into Lecia Deblur (v2.3.2) software. All seven genotypes for each line were processed and imaged at the same time using identical immunostaining, microscope, camera and software settings. Image level normalization, minimum/maximum correction and false coloring were performed using ImageJ (v1.47n). 

### RNA Isolation & cDNA Synthesis

Total RNA was isolated from wing imaginal discs dissected from late third instar larva. 10-15 discs were dissected in SFX media and homogenized in 300 µl TRIzol (Invitrogen) by vortexing for 30 sec. 60 µl chloroform was added and vortexed for 15 s, centrifuged at 12,000g for 10 min at 4°C and the upper aqueous layer precipitated with 150μl isopropanol. Samples were incubated at RT for 10 min and centrifuged at 12,000g for 10 min at 4°C. RNA pellets were washed with 80% EtOH and resuspended in 8.5 μl nuclease-free H_2_O. Genomic DNA was removed by DNAse treatment (*TURBO DNA-Free*, Ambion/Life Technologies) by incubating at 37°C for 20 min. Concentrations and purity were determined using a NanoDrop spectrophotometer, and 500ng of RNA was used for cDNA synthesis using either the iScript^TM^ cDNA synthesis kit containing a blend of oligo dT and random hexamers or the iScript^TM^ Select cDNA synthesis kit with oligo dT primers only (*su*(*Hw*)^e04061^ mutant analysis) (BioRad) for 1 hr at 42°C. 

### Real Time PCR & Data Normalization

qPCR runs were performed on a BioRad iQ5 cycler using iQ SYBR Green Supermix (BioRad) using 1 μl of cDNA and primers specific for *eGFP* and *Rp49*. Both primer sets displayed 99-101% efficiencies. Three biological replicates for each genotype and 3 technical replicates were used and the relative expression was calculated by comparing *eGFP* C_t_ values to *Rp49* C_t_ values following the ΔC_t_ method. For each line, the relative expression for the intact homozygote genotype (P^+^E^+^/P^+^E^+^) was taken to be 1 and the other genotypes normalized accordingly. To derive the final data, the normalized values from all available lines were averaged. Error bars represent standard error of the mean (S.E.M.). Primer sequences are available upon request. 

## Results

### A P-element eGFP Reporter System Engineered to Induce Artificial Mutations in Enhancers and Promoters Shows Insertion Bias To Endogenous Insulators

The somatic pairing dependence required for transvection ultimately derives from the ability of enhancers to act on promoters in close proximity in *trans* when preferential interactions with promoters in *cis* are lost. This feature is supported by the fact that a majority of classical complementing mutant alleles disrupt either the enhancer or the promoter [15]. Our P-element reporter constructs were designed with site-specific recombination sites flanking both these elements in order to selectively delete either the enhancer or the promoter. We chose the *vestigial* boundary enhancer (*vg*BE), which drives expression in a small stripe of cells at the dorsal/ventral boundary in developing wing and haltere imaginal discs [65] and flanked it with *FRT* sites. The minimal hsp70 promoter was flanked by *loxP* sites and followed by the *eGFP* reporter. Two other derivatives of this construct were created by adding either a single *gypsy* insulator upstream of the *vg*BE or two *gypsy* insulators flanking the entire construct (Figure 1A). 

Following microinjection, we recovered 11 independent insertions for the 2-insulator construct, 18 insertions for the 1-insulator construct and 42 insertions for the 0-insulator construct. Analysis of homozygous individuals carrying two copies of the construct from all eleven 2-insulator lines displayed strong eGFP expression in the D/V compartment boundary of the wing discs, particularly along the wing margin within the wing pouch and the hinge region (Figure 1B), whereas only nine 1-insulator line homozygotes and seven 0-insulator line homozygotes displayed detectable eGFP levels, likely the result of position effects [67]. Mapping of these lines revealed a noticeable bias towards the 5' end of genes, transposable elements and other transposon “hotspots”, similar to observations in previous reports [68-70]. Although no correlation with chromatin states/domains were observed [71], nearly all insertions outside of transposons were within 1 Kb of an endogenous protein-bound insulator, particularly those bound by GAF and/or CP190 regardless of whether a *gypsy* insulator was present in the construct (Table 1-3). 

**Table 1:** Chromosome and genomic coordinates for 2-insulator lines.

LINE	CHRM.	GENOMIC COORD.	INSERTION TYPE	HOTSPOT^b^	CHROMATIN TYPE^d^	INSULATOR PROTEINS
PGP-13B	3	3L: 7,127,316	Promoter (*melted*)	*	Blue	CP190, BEAF, Mod, CTCF, GAF, Su(Hw)
PGP-20	2	-^c^	Transposon (*Invader*)	-	-	-
PGP-22A	3	3L: 13,221,600	Promoter (*caps*)	*	Blue	CP190, BEAF, Mod, CTCF, GAF
PGP-23A	3	-^c^	Transposon (*F*)	-	-	-
PGP-25A	3	3R: 22,360,900	Promoter (*CG6490*)	*	Blue/Red	CP190, Mod, GAF
PGP-28^a^	2	2L: 12,018,983	Exon (*CG6734*)	-	Yellow	-
PGP-33A^a^	2	2L: 7,576,480	Promoter (*RapGAP1*)	*	Red	CP190, Mod, GAF, SuHw
PGP-39A^a^	2	2L: 8,989,200	Promoter (*rost*)	-	Red	-
PGP-50A^a^	2	2L: 9,758,467	5' UTR (*zf30C*)	*	Black	CP190, BEAF, Mod, CTCF, GAF
PGP-104A1^a^	2	2L: 20,163,757	Transposon (*Invader*)	-	-	-
PGP-146C2	3	3R: 8,326,193	Intergenic	-	Black	-

^a^ Used for Su(Hw) mutant analysis.

^b^ Transposon hotspot if more than 5 insertions for other P-element or Pbacs were found at this position in FlyBase.

^c^ Not enough flanking sequence recovered to accurately predict insertion position.

^d^ From: Filion et al (2010) Systematic protein location mapping reveals five principal chromatin types in Drosophila cells. Cell 143: 212-224.

**Table 2:** Chromosome and genomic coordinates for 1-insulator lines.

LINE	CHRM.	GENOMIC COORD.	INSERTION TYPE	HOTSPOT^a^	CHROMATIN TYPE^c^	INSULATOR PROTEINS
gi-ve 2	3	3L: 5,177,560	Intron (*shep*)	*	Red	GAF
gi-ve 2A	3	3R: 24,816,510	Intergenic	*	Black	GAF
gi-ve 9A	2	-^b^	Transposon (*gypsy*)	-	-	-
gi-ve 20	3	-^b^	Transposon (*Diver*)	-	-	-
gi-ve 29	3	3R: 7,392,923	Promoter (*sea/fabp*)	*	Red	CTCF, GAF, Su(Hw)
gi-ve 31	2	2R: 6,762,150	Promoter (*CG30015*)	*	Red/Yellow	CP190, BEAF, CTCF, GAF
gi-ve 40	3	3L: 13,470,406	Promoter (s*tv*)	*	Black	GAF
gi-ve 45	3	-^b^	Transposon (*Doc*)	-	-	-
gi-ve 7D	X	X: 13,072,760	Intron (*CG34411*)	-	Black	BEAF, CP190, CTCF

^a^ Transposon hotspot if more than 5 insertions for other P-element or Pbacs were found at this position in FlyBase.

^b^ Not enough flanking sequence recovered to accurately predict insertion position.

^c^ From: Filion et al (2010) Systematic protein location mapping reveals five principal chromatin types in Drosophila cells. Cell 143: 212-224.

**Table 3:** Chromosome and genomic coordinates for 0-insulator lines.

LINE	CHRM.	GENOMIC COORD.	INSERTION TYPE	HOTSPOT^a^	CHROMATIN TYPE^b^	INSULATOR PROTEINS
PNG-10	X	X: 19,671,651	Exon (*vfl*)	-	Blue	-
PNG-11	X	X: 17,793,173	Promoter (*CG32495*)	-	Yellow	CP190, BEAF, CTCF, Mod
PNG-20	2	2R: 17,557,761	5' UTR (*NC2alpha*)	-	Yellow	CP190, BEAF
PNG-44	3	3L: 6,957,768	Promoter (s*gl*)	*	Yellow	CP190, BEAF, GAF
PNG-46	3	3R: 24,953,620	Transposon (*Opus*)	-	-	-
PNG-50	3	3R: 16,886,068	Promoter (*Mvl*)	*	Yellow	CP190, BEAF, CTCF, Mod, GAF
PNG-1C	2	2L: 9,616,828	5' UTR (*GlcAT-S*)	*	Red	CP190, BEAF, CTCF, Mod, GAF

^a^ Transposon hotspot if more than 5 insertions for other P-element or Pbacs were found at this position using FlyBase.

^b^ From: Filion et al (2010) Systematic protein location mapping reveals five principal chromatin types in Drosophila cells. Cell 143: 212-224.

To test the *gypsy* insulator's contribution to transvection, we generated “enhancerless” flies lacking the *vg*BE and “promoterless” flies lacking the *hsp70* promoter for each transgenic line by crossing with flies expressing either Flp or Cre recombinase. Trans-heterozygous progeny for each line were then generated by simply crossing “enhancerless” and “promoterless” homozygotes, allowing for a direct readout of transvection effects. We hypothesized that loss of communication between the *vg*BE and the *hsp70* promoter in *cis* due to promoter removal could be restored in *trans* by stable interactions between *gypsy* components, inducing eGFP expression from the “enhancerless” homolog along the D/V boundary of the wing disc (Figure 1B & 1C). 

### Homolog Pairing Increases Reporter Levels Independently of gypsy

**Figure 2:**
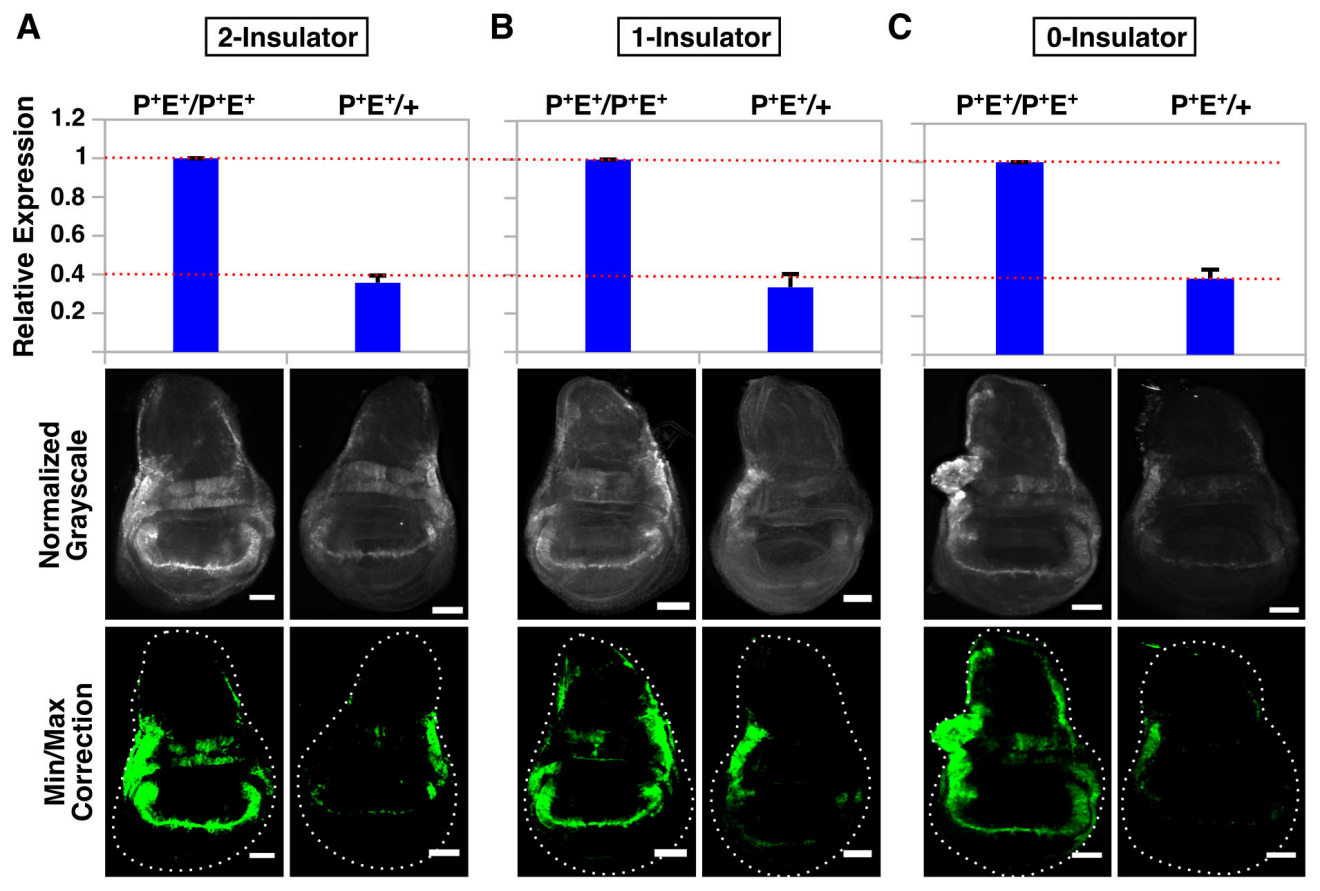
Pairing can stimulate *eGFP* transcription more than two-fold independently of *gypsy* presence. QPCR analysis (top graph) and immunostaining (bottom panels) of wing discs from intact homozygotes (P^+^E^+^/P^+^E^+^) and intact hemizygotes (P^+^E^+^/+) for 2-insulator (A), 1-insulator (B) and 0-insulator (C) lines. For microscopy, images were normalized to P^+^E^+^/P^+^E^+^ for each respective line and minimum/maximum level corrections were applied equally to both genotypes using ImageJ and false-colored green. Error bars represent standard error of the mean (S.E.M) and scalebars are 50 μm.

Previous studies in wing imaginal disc have suggested that pairing synapsis between homologous chromosomes can stimulate enhanced transcription of *Ubx* from both homologs [83]. We began by examining reporter levels in homozygous and hemizygous flies carrying an intact (i.e., non-deleted enhancer/promoter) construct. Immunostaining of wing discs revealed a significant decrease in the amount of GFP protein in hemizygous larvae carrying the construct on only one homolog compared to homozygous larvae carrying a copy on each homolog, a pattern independent of *gypsy* presence (Figure 2A-2C). In terms of dosage, the expression level of *GFP* in homozygotes would be expected to be twice the amount observed in hemizygotes, particularly if levels were influenced solely by enhancer-promoter communication in *cis*. Using qPCR, we measured the levels of *GFP* expression and found that transcript levels were reduced 2.5-3X in hemizygotes compared to homozygotes, suggesting that pairing in *trans* can stimulate increased transcription (Figure 2A-2C) in agreement with previous findings [83]. However, this behavior was not significantly influenced by the *gypsy* insulator, as the 2.5x, 3x, and 2.8x reduction observed for the 0-, 1- and 2-insulator hemizygotes, respectively, rules out any synergistic effect that would be expected if this element contributed to pairing. 

### The vgBE Can Drive Reporter Expression In The Absence of a Functional Promoter

**Figure 3:**
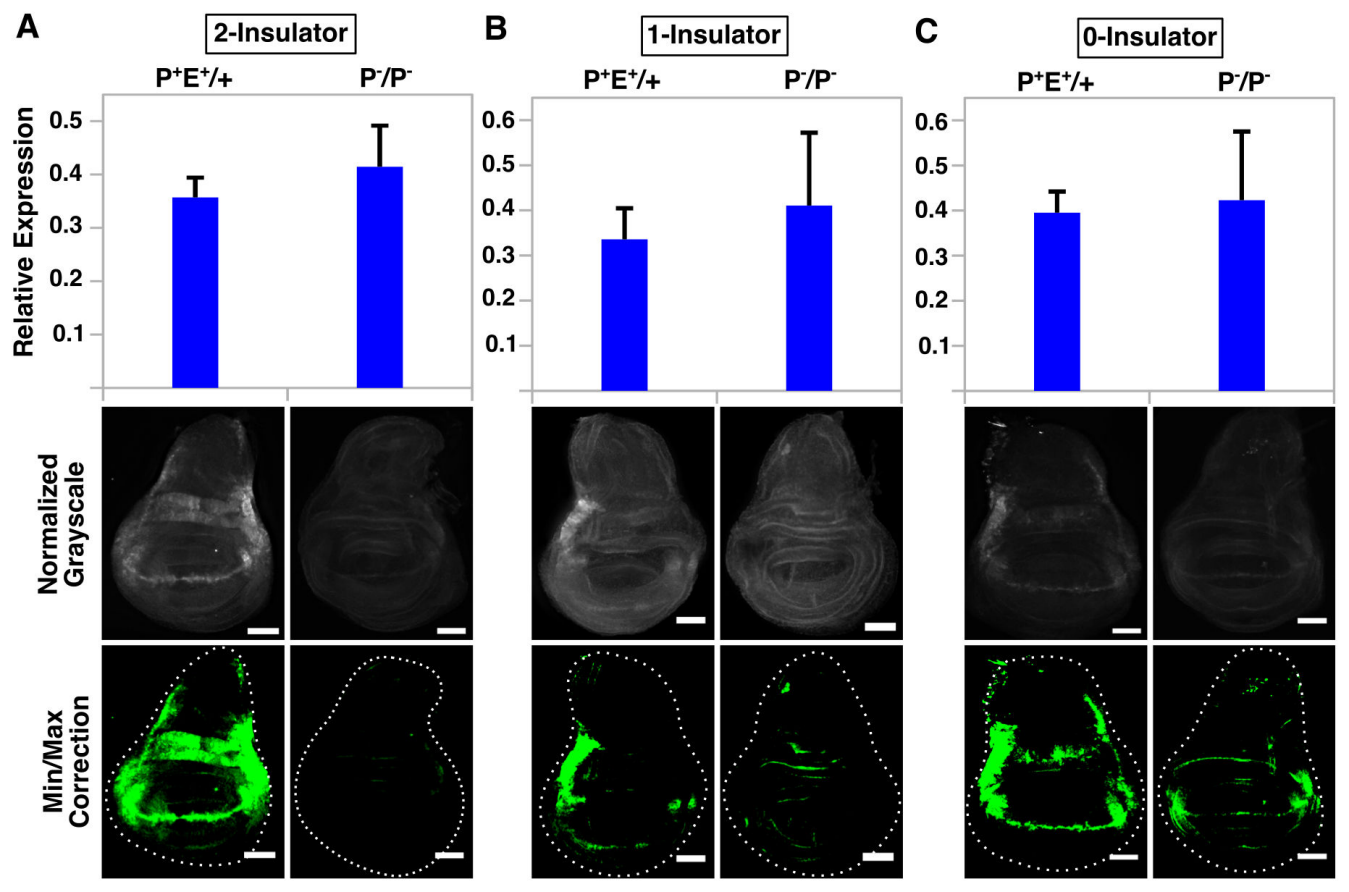
The *vg*BE can drive *eGFP* reporter expression in the absence of a functional promoter but this does not correlate with GFP protein levels. QPCR analysis (top graph) and immunostaining (bottom panels) of wing discs from intact hemizygotes (P^+^E^+^/+) and “promoterless” homozygotes (P-/P^-^) for 2-insulator (A), 1-insulator (B) and 0-insulator (C) lines. For microscopy, images were normalized to P^+^E^+^/+ for each respective line and minimum/maximum level corrections were applied equally to both genotypes using ImageJ and false-colored green. Error bars represent standard error of the mean (S.E.M) and scalebars are 50 μm.

Next, we tested whether *gypsy* might influence enhancer-promoter communication in *trans* by removing either the *vg*BE or the *hsp70* minimal promoter to generate “enhancerless” and “promoterless” lines (Figure S1A) and combining them to create trans-heterozygous individuals. However, qPCR revealed a large amount of *GFP* transcript present in promoterless lines, with levels in the promoterless homozygote (P^-^/P^-^) equal or greater to the levels observed in the non-deleted hemizygote (P^+^E^+^/+). This pattern of expression was consistent in every single line examined, independent of *gypsy* insulator presence (Figure 3A-3C), suggesting that the *vg*BE can drive expression in the absence of a functional promoter. We suspected that perhaps *vg*BE's proximity to the coding region of *GFP* (~183 bp after *hsp70* promoter deletion) might explain this result, as any RNA Pol II recruited to the *vg*BE and able to find a suitable transcription start site (TSS) might be able to generate a transcript. We found two TSSs in potential promoters within the *vg*BE using a neural network prediction algorithm (minimum promoter score=0.8) [72]; however, using qPCR primers designed to only measure mRNAs arising from these TSSs, we could not detect a sufficient amount of transcripts to fully account for the total pool of *GFP* mRNA (Figure S1B). It is worth noting that our *GFP* qPCR primers are located at the 3' end of the transcript (within 130 bp of the stop codon), which would fail to distinguish whether other cryptic TSSs located downstream of our *vg*BE qPCR primers might be utilized and therefore contribute to the pool of transcripts as well. 

Interestingly, *GFP* transcript levels as measured by qPCR did not correlate with the amount of GFP protein. Analysis of wing discs stained with α-GFP revealed only a small amount of signal, barely above background levels, or no signal at all along the DV boundary within the wing pouch and hinge in promoterless homozygotes (P^-^/P^-^). Non-deleted hemizygotes (P^+^E^+^/+), on the other hand, displayed strong signal along the boundary (Figure 3A-3C). This pattern was consistent in all lines examined and independent of *gypsy* insulator presence. Background levels of expression in other parts of the wing disc were similar between the two genotypes, ruling out the possibility that misexpression by other regulatory elements might contribute to the large amount of *GFP* transcript in promoterless individuals. Although we have not measured mRNA or protein stability directly in these lines, it should be noted that cDNA generated using two different priming methods (a mix of random hexamers and oligo dT primers or oligo dT primers only) gave identical results (Figure S1C), suggesting that our results are not due to primer bias during cDNA synthesis and that the majority of transcripts being measured were polyadenylated. Taken collectively, our results suggest that although the *vg*BE can drive reporter expression in the absence of a functional promoter, many of these transcripts do not give rise to functional protein. 

### Two *gypsy* Insulators Can Facilitate Enhancer-Promoter Communication in *Trans*

**Figure 4:**
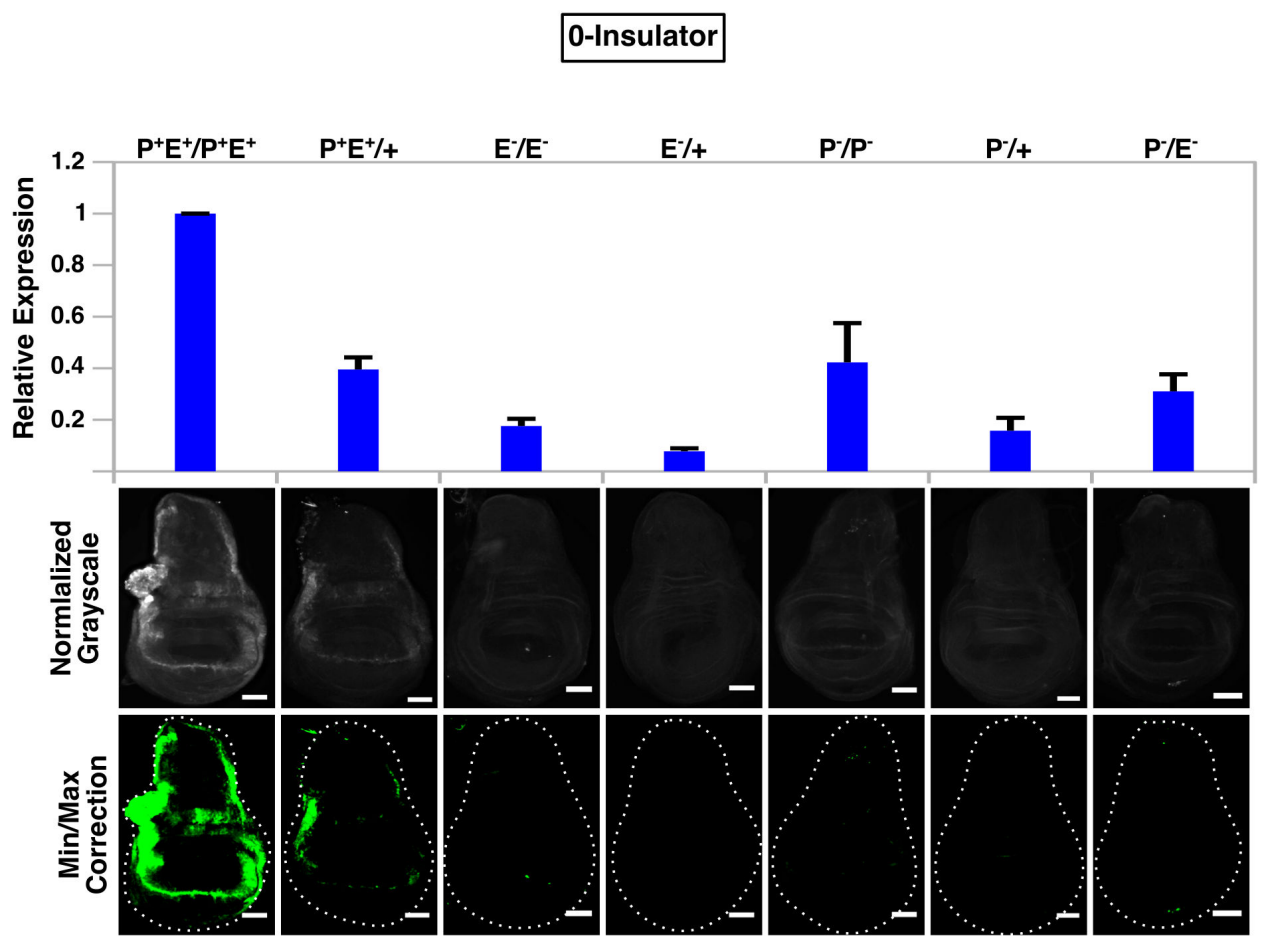
Absence of *gypsy* fails to promote enhancer-promoter communication in *trans*. QPCR analysis (top graph) and immunostaining (bottom panels) of wing discs from all seven 0-insulator genotypes. For microscopy, all images were normalized to P^+^E^+^/P^+^E^+^ and minimum/maximum level corrections were applied equally to all genotypes using ImageJ and false-colored green. Error bars represent standard error of the mean (S.E.M) and scalebars are 50 μm.

**Figure 5:**
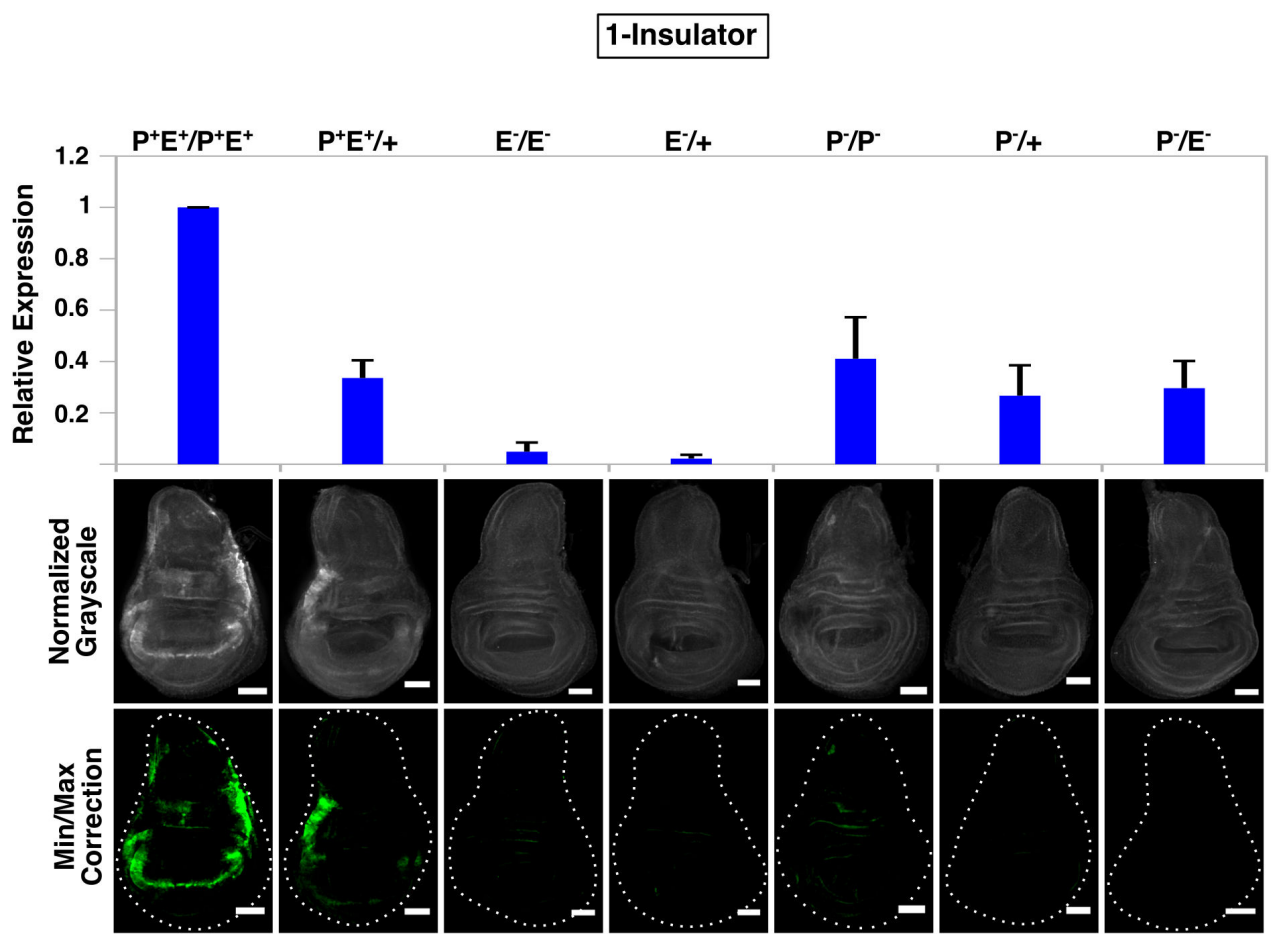
A single *gypsy* fails to promote enhancer-promoter communication in *trans*. QPCR analysis (top graph) and immunostaining (bottom panels) of wing discs from all seven 1-insulator genotypes. For microscopy, all images were normalized to P^+^E^+^/P^+^E^+^ and minimum/maximum level corrections were applied equally to all genotypes using ImageJ and false-colored green. Error bars represent standard error of the mean (S.E.M) and scalebars are 50 μm.

**Figure 6:**
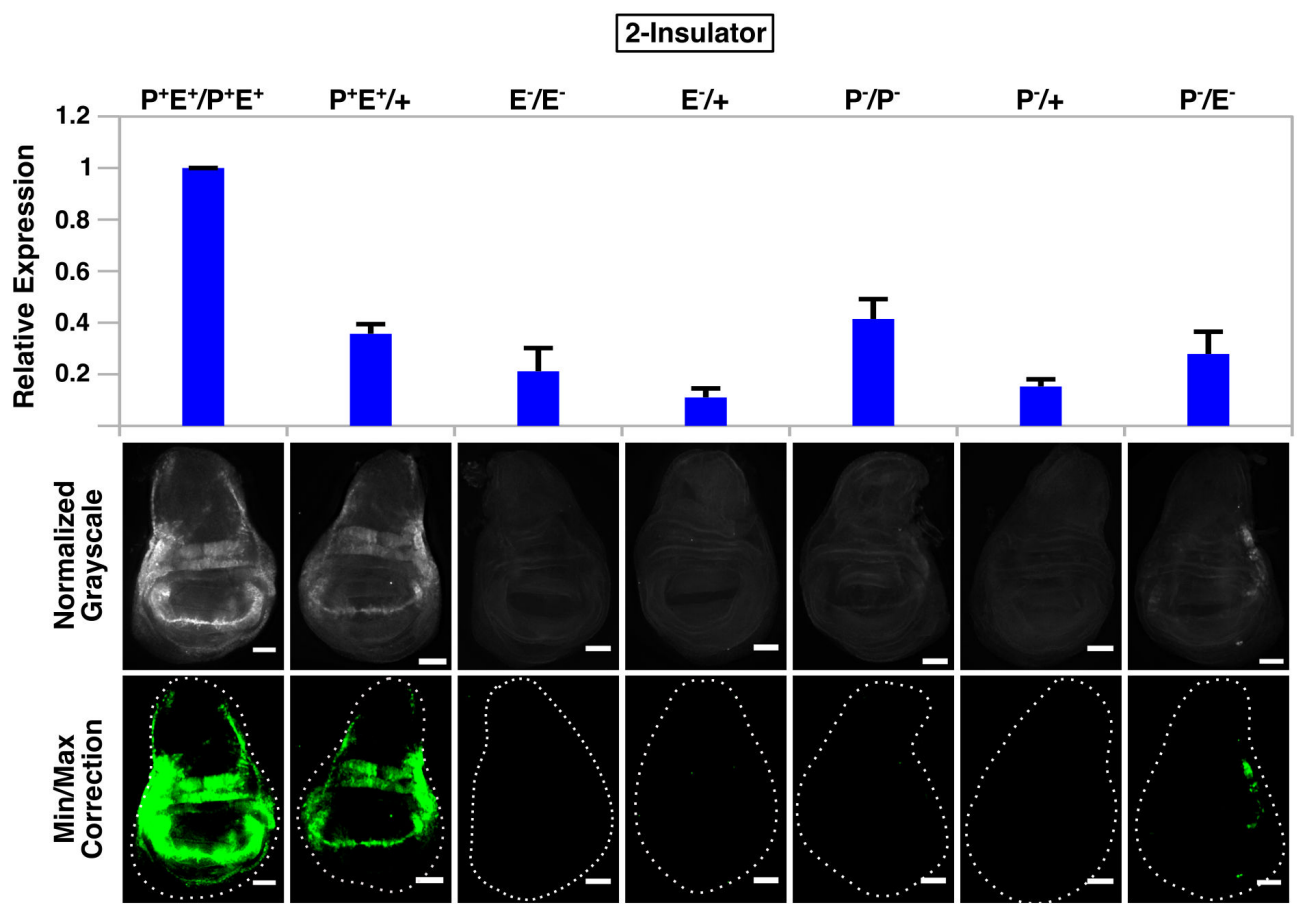
Flanking *gypsy* insulators promote enhancer-promoter communication in *trans*. QPCR analysis (top graph) and immunostaining (bottom panels) of wing discs from all seven 2-insulator genotypes. For microscopy, all images were normalized to P^+^E^+^/P^+^E^+^ and minimum/maximum level corrections were applied equally to all genotypes using ImageJ and false-colored green. Error bars represent standard error of the mean (S.E.M) and scalebars are 50 μm.

We next determined whether *gypsy* could promote enhancer-promoter communication in *trans* by staining wing discs with α-GFP from larvae containing a single functional enhancer on one homolog and a single functional promoter on the other. GFP signal was barely above background or undetectable along the wing margin and hinge region in 0-insulator and 1-insulator trans-heterozygotes (P^-^/E^-^) (Figure 4 and Figure 5). However, moderate levels of GFP were observed in 2-insulator trans-heterozygotes, with most of the expression concentrated in the hinge region as staining along the wing margin was considerably weaker and variegated (Figure S2). No signal was observed for either the promoterless (P^-^/+) or enhancerless (E^-^/+) hemizygote, and although a small amount of protein could be detected in the promoterless homozygote (P^-^/P^-^), the GFP signal was much stronger in the trans-heterozygote (P^-^/E^-^) (Figure 6). Taken collectively, these data suggest that the presence of two flanking *gypsy* insulators is sufficient to support enhancer-promoter communication in *trans*. Strangely, however, our qPCR results did not agree with our image analysis—we were unable to detect the additional transcripts that should have been present in the 2-insulator trans-heterozygote. Instead, the 2-insulator, 1-insulator and 0-insulator lines all displayed the same behavior: the trans-heterozygote expression level was always the sum of the promoterless (P^-^/+) and enhancerless (E^-^/+) hemizygote expression levels, a finding that would be expected if homologs were acting independently of one another (i.e., in cis) and transvection was absent. However, given the caveats of qPCR analysis of our system described earlier (Figure 3), we believe that our immunostaining and subsequent image analysis is the most accurate reflection of whether trans-interactions are occurring or not and therefore conclude that two *gypsy* insulators are sufficient to mediate transvection. 

### Mutations in Su(Hw) Reduce Reporter Levels in a Pairing-Independent manner

Finally, to confirm that the *gypsy* insulator plays a direct role in mediating transvection, we combined a subset of our 2-insulator lines into a genetically null *su*(*Hw*)^*e04061*^ mutant background and measured GFP reporter levels by both immunostaining and qPCR. Su(Hw) was the first protein identified shown to be critical for the insulator properties of *gypsy* and has been shown to be required for somatic pairing in embryos [63,73-75], suggesting that Su(Hw) and other insulator proteins distributed throughout the genome might contribute to transvection in a global manner. If the reduction in transcription levels observed in mutants is attributable to pairing influences, then we would expect that only homozygous individuals carrying the reporter on each homolog would be affected, as hemizygous individuals lacking a suitable pairing region on the opposite homolog should not be affected by loss of such pairing. Immunostaining and qPCR analysis of mutant wing discs revealed a significant decrease in reporter expression for both homozygotes and hemizygotes (Figure S3A). Since transcription does not appear to be globally perturbed in a *su*(*Hw*)^*e04061*^ background (Figure S3B), it is likely that the reduced reporter expression we observe is due to position effects resulting from the failure of the flanking *gypsy* insulators to prevent repressive chromatin from spreading into the reporter locus in *cis* due to loss of Su(Hw) [67], rather than a reduction or loss of homolog pairing. 

## Discussion

In this work we have utilized a transgenic reporter engineered to induce artificial mutations in the *vg*BE enhancer and *hsp70* promoter in order to test the role of the *gypsy* insulator in the phenomenon of transvection. We find that although pairing does appear to modestly increase the amount of *eGFP* transcript arising from intact constructs containing functional regulatory elements in *cis* on each homolog, this behavior is not dependent on the *gypsy* insulator. However, flanking *gypsy* insulators are required for transvection when enhancerless and promoterless homologs were combined in *trans*. Interestingly, we also found that the *vg*BE can drive transcription of the reporter in the absence of a functional promoter. From these findings, we conclude that *gypsy* can support interactions in *trans* in a dose-dependent manner, although we cannot rule out that other insulator binding sites may contribute to pairing in cooperation with other factors at specific genomic locations, depending on the local chromatin landscape. 

 Recent reports have utilized a reporter scheme analogous to ours, whose main advantage lies in the fact that transvection can be tested in a tightly controlled manner using fluorophores that allow for single-cell analysis of enhancer-promoter communication in *trans*, as opposed to phenotypic analysis of whole animals [10,36]. However, unlike those reports, which took advantage of the φC31 integration system to test only a handful of characterized integration sites in the genome [76], we utilized a P-element transformation method to integrate our reporters into a number of different regions of the genome, particularly repetitive sequences, transposon hotspots and the 5' end of genes [68-70] in order to provide a more global analysis of transvection effects. 

Image analysis strongly supports the conclusion that the 2-insulator construct can support transvection, as moderate amounts of GFP along the DV boundary, primarily in the hinge region, was readily observable at levels much higher than either promoterless (P^-^/+) or enhancerless (E^-^/+) hemizygote, which displayed no signal whatsoever. Perhaps most tellingly, the amount of signal in the promoterless homozygotes (P^-^/P^-^) was much lower than the trans-heterozygote, suggesting that pairing a functional enhancer with a functional promoter in *trans* can positively stimulate transcription above background levels if aided by two *gypsy* insulators. However, using qPCR we were unable to detect the expected increase in transcripts in the 2-insulator trans-heterozygous lines as compared to either the 1- or 0-insulator lines. We have shown that transcript levels, as measured by qPCR, do not accurately reflect the amount of protein present in the promoterless genotypes—in all lines, regardless of insulator presence, transcript levels were consistently and reproducibly equal to or higher in all promoterless homozygotes (P^-^/P^-^) as compared to non-deleted hemizygotes (P^+^E^+^/+), despite weak levels of GFP protein staining for P^-^/P^-^ and high levels for P^+^E^+^/+. The most likely explanation for this observation is that the *vg*BE enhancer in the promoterless transgene can activate transcription using cryptic promoters that lead to a pool of *eGFP* transcripts that remain untranslated or that are translated into non-functional protein. Therefore, we argue that our immunostaining and image analysis are the most appropriate metric by which to evaluate *gypsy* insulator function in transvection.

Such spurious transcripts might also explain why we do not observe an increase in the relative number of total transcripts in the 2-insulator transvection lines as compared to the 1- and 0-insulator lines. Rather, perhaps it is simply a shift in the relative ratio of the different types of messages. For simplicity, one could imagine two types of *GFP* transcripts that differ in their 5' ends: one that gives rise to a functional GFP protein and the other that gives rise to a non-functional protein. In this case, the functional transcript would only arise from the homolog containing the intact promoter (which would only be possible in *trans*) whereas the non-functional transcript would arise from the promoterless homolog in *cis*. For 1- and 0-insulator trans-heterozygotes, most *eGFP* transcripts would be the non-functional message driven in *cis* due to the failure of the *vg*BE to stably communicate with the functional promoter in *trans*. However, in 2-insulator trans-heterozygous individuals, this proportion would be reversed, with most *eGFP* transcripts being functional as a result of stable communication in *trans* between the *vg*BE and functional promoter due to the influence of the flanking *gypsy* insulators. Note that this argument is only valid if we assume that the total output of the *vg*BE is equal regardless of whether *cis* or *trans* interactions predominate and only if it is acting in either conformation at a given point in time, not both simultaneously. This idea is supported by the fact that although regulatory elements prefer to act in *cis* [22,77-80], competition between promoters for a single enhancer ultimately dictates whether the enhancer functions primarily in *cis* or in *trans* within the same cell [10].

**Figure 7:**
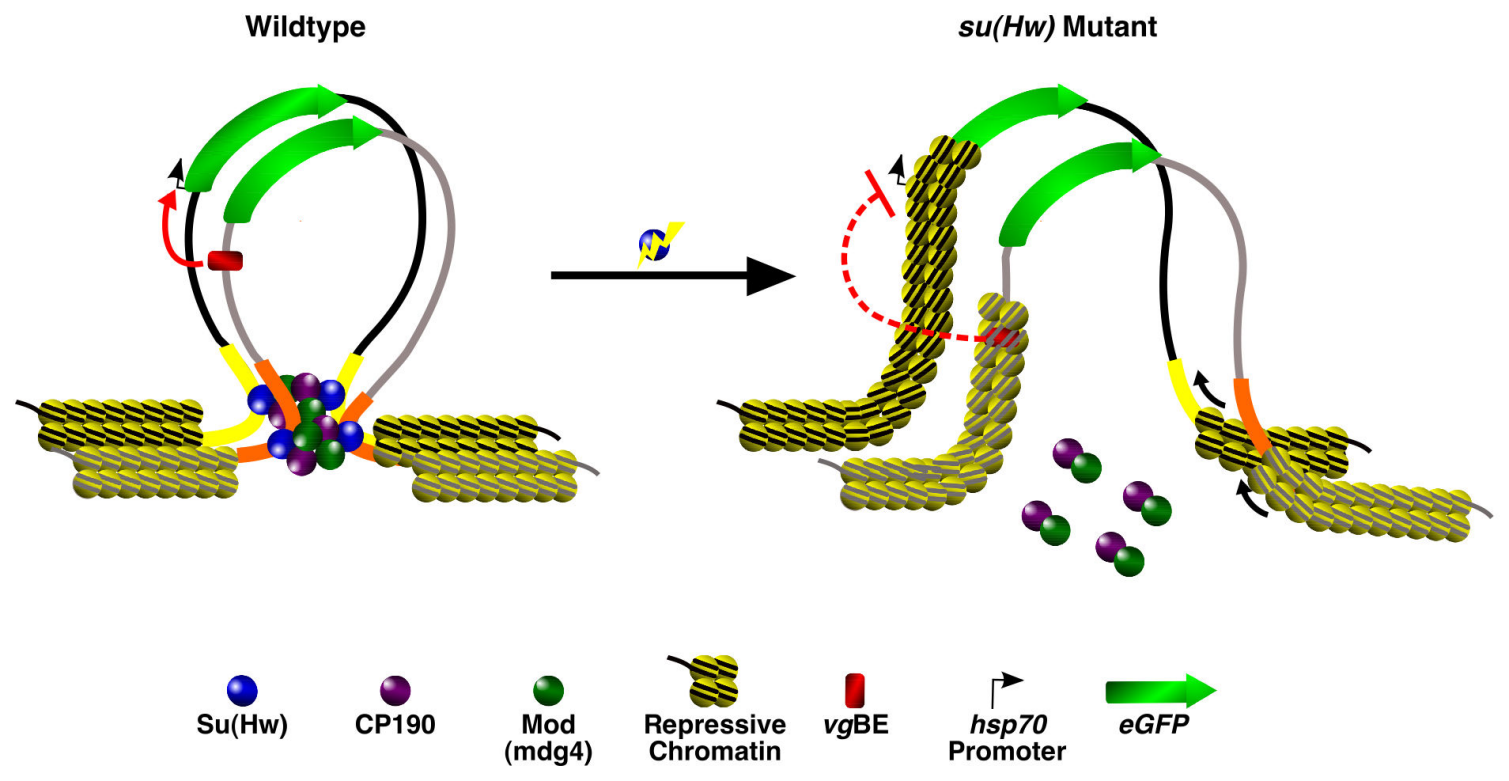
A model for *gypsy* insulator function in transvection. Looping in *cis* between flanking insulators in a wildtype background prevents silencing of regulatory elements located on opposite homologs. Mutations in *su*(Hw) disrupt *cis* looping contacts and alter the local chromatin landscape to a more repressive state, preventing stable communication between regulatory elements even if pairing between homologs is maintained.

Finally, why do two flanking *gypsy* insulators, but not a single upstream *gypsy* insulator, support transvection? One might assume that if *gypsy* contributes to homolog pairing, then a single insulator located just upstream of the enhancer and promoter would still be capable of ensuring that those two elements remain in close proximity in *trans*. However, we argue that the most critical determinant is chromatin structure itself—it is widely accepted as a key regulator of transcription in *cis*, so the same principles would also apply in *trans*. Even if pairing were to bring enhancers and promoters in close proximity, the underlying chromatin must still be permissible in order for transcription to occur. Insulators were originally identified based on their ability to buffer the effects of surrounding chromatin influences (i.e., position effects) on transgene expression [81,82], and regardless of the mechanism by which insulators accomplish this task (chromatin looping, etc.) it is likely that the transvection we observe is due to the flanking insulators establishing a permissive chromatin environment favorable for transcription. The single *gypsy* insulator, on the other hand would not be able to establish the same environment and therefore even if pairing were established by other elements, transcription would still be unlikely to occur given the lack of a suitable chromatin landscape (Figure 7). Our *su*(*Hw*) mutant data supports this hypothesis, as significant reductions in GFP expression were observed in both homozygotes and hemizygotes, highlighting the importance of chromatin structure on transgene expression regardless of pairing influences. Our findings, along with a number of other studies linking chromatin proteins to transvection [25,32,48-52] and the failure of other studies to observe transvection except when their reporters were located in defined PhiC31 genomic sites that are highly permissible to transcription [36,67], suggests that chromatin itself is the master regulator of this phenomenon. 

## Supporting Information

Figure S1 **Confirmation of *vg*BE and *hsp70* promoter deletion for a single representative 2-insulator, 1-insulator and 0-insulator line.** Schematic (right) shows position of PCR primers (red arrowheads) and the size of each respective element (A). Schematic showing potential position of cryptic transcription start sites (TSSs) in the *vg*BE (red triangles), the distance between the *vg*BE and *eGFP* start codon following promoter removal and the position of test primers (tan and blue arrows) used for qPCR. Graphs showing transcript levels based on these primers for a single “promoterless” (P^-^/P^-^) representative from 2-insulator, 1-insulator and 0-insulator lines are shown (B). qPCR analysis of *eGFP* transcript levels reverse-transcribed using either a mixture of random hexamers+oligo dT primers or oligo dT primers alone for cDNA synthesis for the indicated 2-insulator genotypes (C). All error bars represent the standard error of the mean (S.E.M.).
(TIFF)

Figure S2 **2-insulator trans-heterozygote (P^-^/E^-^ ) GFP protein is enriched primarily in the hinge with a variegated pattern of weak expression in the wing margin.** Shown is a collection of 8 different wing discs from a representative 2-insulator P^-^/E^-^ line (grayscale images) with magnified panels below showing a closeup of the yellow boxed region false-colored in green. All grayscale levels were normalized equally, while min/max corrections to magnified panels were performed separately for each disc using ImageJ. Scalebars in grayscale panels are 50 μm and 10 μm in magnified panels.
(TIFF)

Figure S3 **Mutations in *su*(*Hw*) decrease reporter level expression in a pairing-independent manner.** QPCR analysis (top graph) and immunostaining (bottom panels) of wing discs from all seven 2-insulator genotypes in a TM6B-balanced or *su*(*Hw*)^*e04061*^ mutant background. For microscopy, all grayscale images were normalized to the balanced P^+^E^+^/P^+^E^+^, while minimum/maximum level corrections were applied equally to both balanced and *su*(*Hw*)^*e04061*^ backgrounds based on each individual *reporter* *genotype*. Thus, each of the 14 genotypes irrespective of genetic background are directly comparable in the normalized grayscale panels, while only a single genotype (such as P^-^/P^-^) is directly comparable between backgrounds in the minimum/maximum corrected panels (A). qPCR analysis of *SUMO, Actin* and *JNK* (*bsk*) expression levels in a balanced or *su*(*Hw*)^*e04061*^ background (B). Error bars represent standard error of the mean (S.E.M) and scalebars are 50 μm.
(TIFF)
