# Supplementary figures and images for: The Drosophila gypsy Insulator Supports Transvection in the Presence of the vestigial Enhancer
Source: PLoS One. 2013 Nov 13;8(11):e81331. doi: 10.1371/journal.pone.0081331 (PMC3827471; doi:10.1371/journal.pone.0081331)

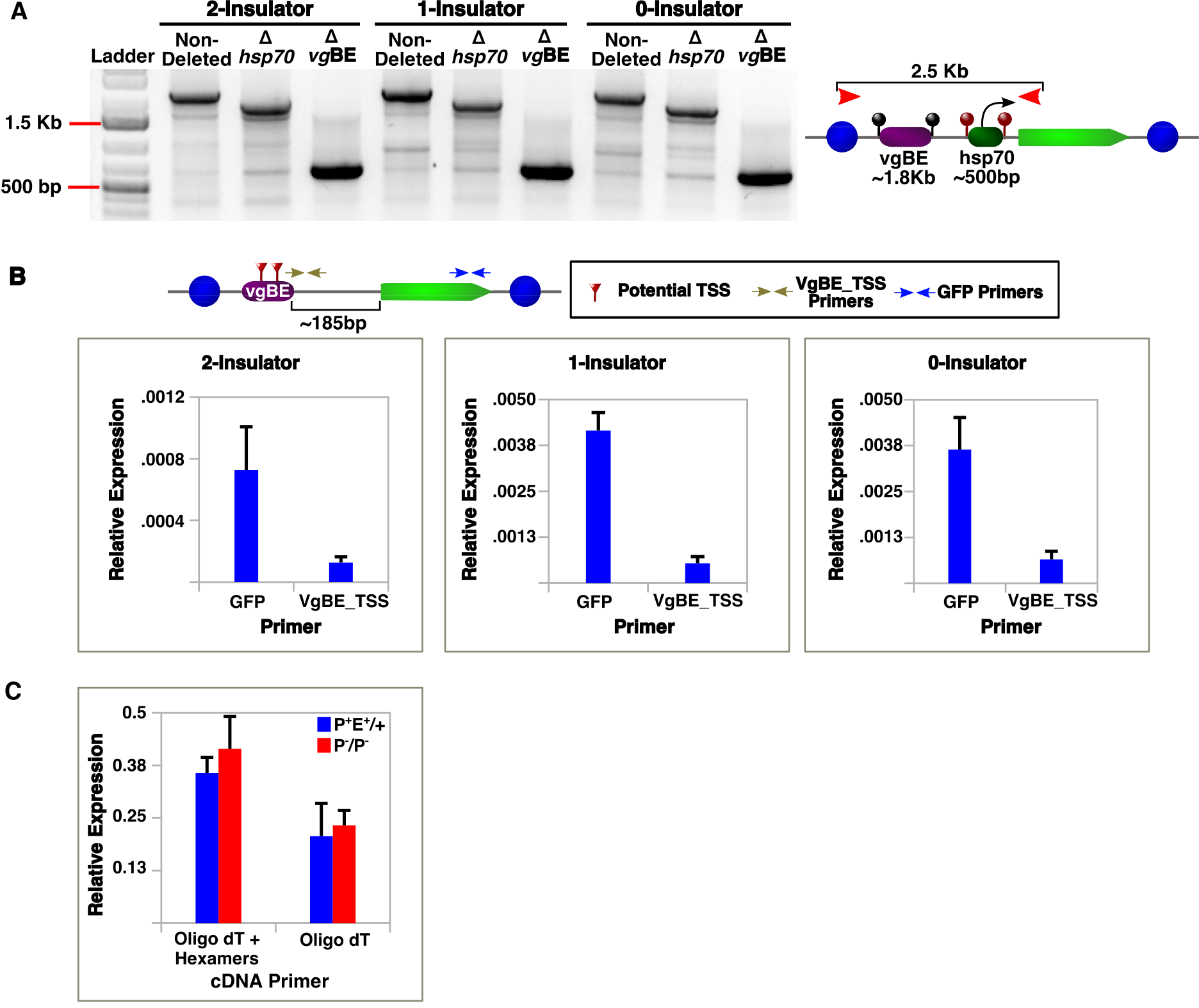

Supplement: Figure S1 — Confirmation of vgBE and hsp70 promoter deletion for a single representative 2-insulator, 1-insulator and 0-insulator line. Schematic (right) shows position of PCR primers (red arrowheads) and the size of each respective element (A). Schematic showing potential position of cryptic transcription start sites (TSSs) in the vgBE (red triangles), the distance between the vgBE and eGFP start codon following promoter removal and the position of test primers (tan and blue arrows) used for qPCR. Graphs showing transcript levels based on these primers for a single “promoterless” (P-/P-) representative from 2-insulator, 1-insulator and 0-insulator lines are shown (B). qPCR analysis of eGFP transcript levels reverse-transcribed using either a mixture of random hexamers+oligo dT primers or oligo dT primers alone for cDNA synthesis for the indicated 2-insulator genotypes (C). All error bars represent the standard error of the mean (S.E.M.). (TIFF) [file pone.0081331.s001.tiff]

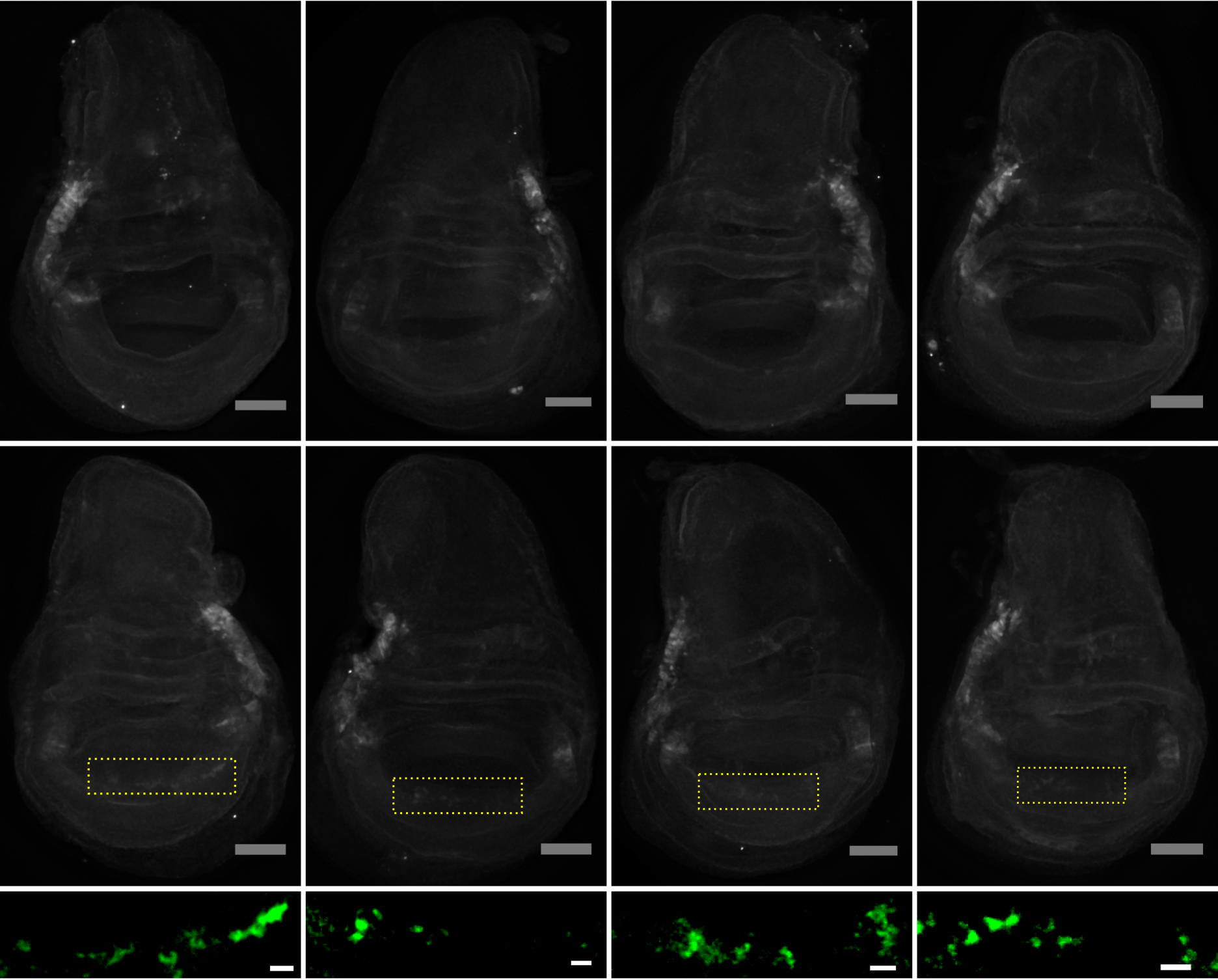

Supplement: Figure S2 — 2-insulator trans-heterozygote (P-/E- ) GFP protein is enriched primarily in the hinge with a variegated pattern of weak expression in the wing margin. Shown is a collection of 8 different wing discs from a representative 2-insulator P-/E- line (grayscale images) with magnified panels below showing a closeup of the yellow boxed region false-colored in green. All grayscale levels were normalized equally, while min/max corrections to magnified panels were performed separately for each disc using ImageJ. Scalebars in grayscale panels are 50 μm and 10 μm in magnified panels. (TIFF) [file pone.0081331.s002.tiff]

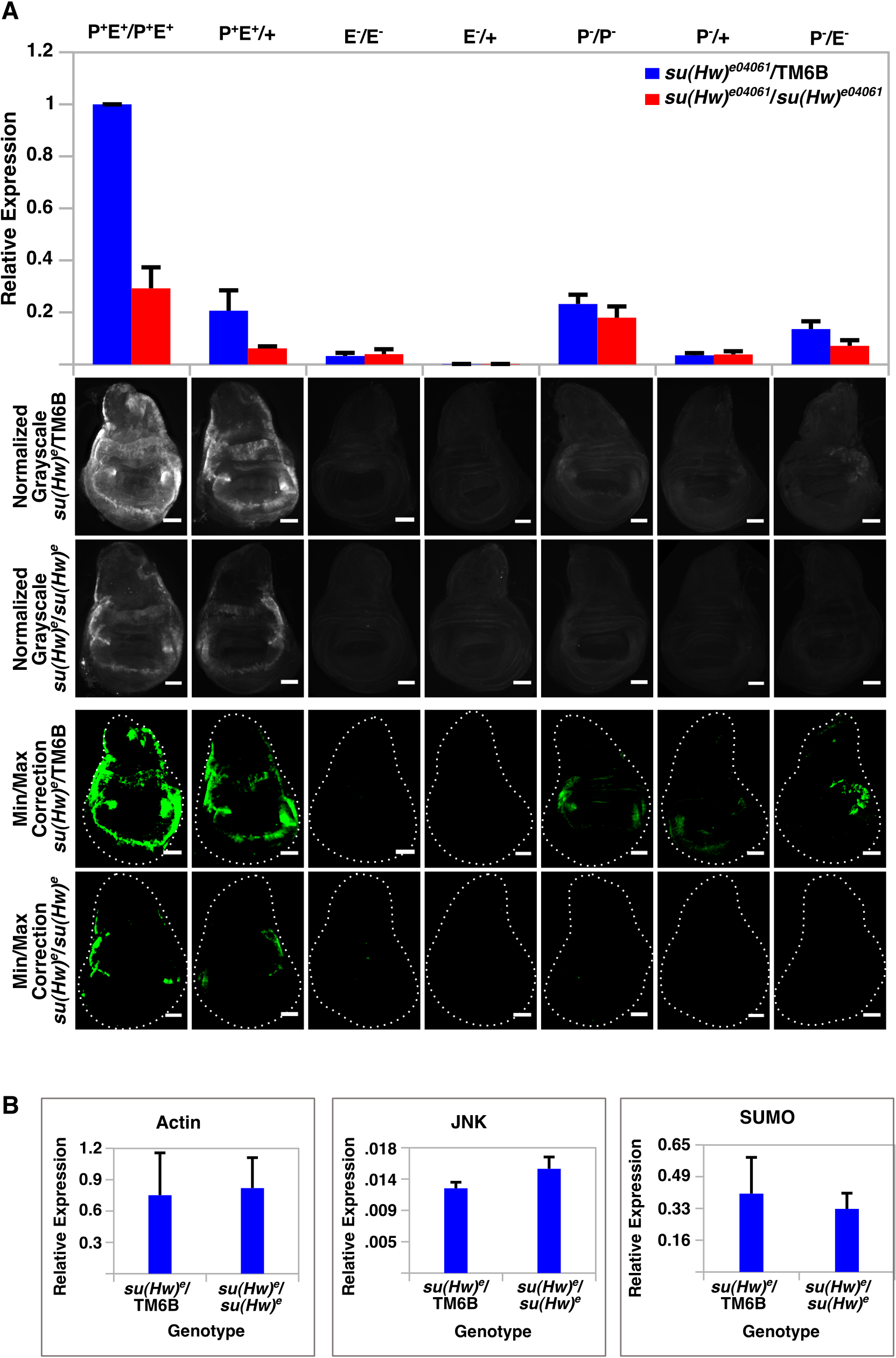

Supplement: Figure S3 — Mutations in su(Hw) decrease reporter level expression in a pairing-independent manner. QPCR analysis (top graph) and immunostaining (bottom panels) of wing discs from all seven 2-insulator genotypes in a TM6B-balanced or su(Hw)e04061 mutant background. For microscopy, all grayscale images were normalized to the balanced P+E+/P+E+, while minimum/maximum level corrections were applied equally to both balanced and su(Hw)e04061 backgrounds based on each individual reporter genotype. Thus, each of the 14 genotypes irrespective of genetic background are directly comparable in the normalized grayscale panels, while only a single genotype (such as P-/P-) is directly comparable between backgrounds in the minimum/maximum corrected panels (A). qPCR analysis of SUMO, Actin and JNK (bsk) expression levels in a balanced or su(Hw)e04061 background (B). Error bars represent standard error of the mean (S.E.M) and scalebars are 50 μm. (TIFF) [file pone.0081331.s003.tiff]
